# The Merging of Two Dynasties—Identification of an African Cotton Leaf Curl Disease-Associated Begomovirus with Cotton in Pakistan

**DOI:** 10.1371/journal.pone.0020366

**Published:** 2011-05-26

**Authors:** Muhammad Nouman Tahir, Imran Amin, Rob W. Briddon, Shahid Mansoor

**Affiliations:** Agricultural Biotechnology Division, National Institute for Biotechnology and Genetic Engineering, Faisalabad, Pakistan; Institute of Infectious Disease and Molecular Medicine, South Africa

## Abstract

Cotton leaf curl disease (CLCuD) is a severe disease of cotton that occurs in Africa and Pakistan/northwestern India. The disease is caused by begomoviruses in association with specific betasatellites that differ between Africa and Asia. During survey of symptomatic cotton in Sindh (southern Pakistan) *Cotton leaf curl Gezira virus* (CLCuGV), the begomovirus associated with CLCuD in Africa, was identified. However, the cognate African betasatellite (Cotton leaf curl Gezira betasatellite) was not found. Instead, two Asian betasatellites, the CLCuD-associated Cotton leaf curl Multan betasatellite (CLCuMB) and Chilli leaf curl betasatellite (ChLCB) were identified. Inoculation of the experimental plant species *Nicotiana benthamiana* showed that CLCuGV was competent to maintain both CLCuMB and ChLCB. Interestingly, the enations typical of CLCuD were only induced by CLCuGV in the presence of CLCuMB. Also in infections involving both CLCuMB and ChLCB the enations typical of CLCuMB were less evident. This is the first time an African begomovirus has been identified on the Indian sub-continent, highlight the growing threat of begomoviruses and particularly the threat of CLCuD causing viruses to cotton cultivation in the rest of the world.

## Introduction

Cotton leaf curl disease (CLCuD) is a serious disorder of cultivated cotton that is characterised by greening (affected plants, at least early during infection, appear darker green that unaffected plants), leaf curling, vein darkening, vein swelling, enations and production of cup-shapped leaf-like structures on the undersides of leaves. The disease has been reported from a number of countries across Africa and from southern Asia, specifically Pakistan and northwestern India. In Africa CLCuD was first reported 1912 from Nigeria, affecting *Gossypium barbadense,* and then from Sudan in 1924 and subsequently from Tanzania in 1926 [1]. These early studies showed the disease to be of virus etiology and to be transmitted by the whitefly *Bemisia tabaci*. Losses to cotton cultivation in the Sudan were ultimately overcome by the introduction of resistant cotton varieties. In Pakistan CLCuD was a minor, sporadic problem until 1986, when a plot of a newly introduced cotton variety (*G. hirsutum*) became infected. In subsequent years it became epidemic spreading to almost all cotton growing regions of Pakistan and into adjoining areas of India. Losses to the economy of Pakistan during this period were enormous; estimated at US$5 billion between 1992 and 1997. During the late 1990s losses were gradually reduced, returning Pakistan's cotton output to above pre-epidemic levels, by the introduction of CLCuD-resistant cotton varieties. Unfortunately, in 2001, all previously resistant cotton varieties began to show the typical symptoms of CLCuD, indicating that the resistance had been overcome [2]. At this time, Pakistan and northwestern India are severely affected by the disease. Although thought to be caused by viruses, the etiology of CLCuD was not finally resolved until the early 21^st^ Century. For both southern Asia [3] and north Africa [4], [5] the disease was shown to be caused by viruses of the genus *Begomovirus* (family *Geminiviridae*) in association with a newly identified class of satellites, the betasatellites [6].

Geminiviruses are insect-transmitted viruses with single-stranded (ss)DNA genomes which are encapsidated in characteristic twinned icosahedral particles from which they derive their name. The family *Geminiviridae* encompasses four genera (*Mastrevirus*, *Topocuvirus*, *Curtovirus* and *Begomovirus*) and viruses are assigned to these based on genome organization, host range and insect vector [7], [8]. The viruses of the genus *Begomovirus* are the most numerous and economically the most destructive. They are transmitted by a single species of whitefly (*Bemisia tabaci*) and have genomes that consist of either two components (known as DNA-A and DNA-B) or a single component (homologous to the DNA-A component of the bipartite viruses). Each component is 2.6–2.8 kb in size, replicates in the nucleus and is transcribed bidirectionally from a non-coding region which also contains a hairpin structure (which within the loop contains the sequence TAATATTAC known as the nonanucleotide motif) that forms part of the origin of replication. All begomoviruses native to the New World have bipartite genomes. In the Old World, although a few bipartite begomoviruses have been identified, the vast majority are monopartite and most of these associate with a class of ssDNA satellites known as betasatellites as well as satellite like molecules known as alphasatellites.

Satellites are defined as viruses or nucleic acids that depend on a helper virus for their replication but lack extensive nucleotide sequence homology to the helper virus and are dispensable for its proliferation [9]. Betasatellite are small, approximately half the size of a begomovirus component (∼1350 nt), ssDNA molecules that are often involved in induction of typical symptoms in plant hosts [6]. Betasatellites (previously known as DNA β) depend on their helper begomoviruses for replication and movement within and between plants. Although highly diverse, betasatellites have a conserved structure consisting of an adenine rich region, a sequence conserved between all betasatellites (the satellite conserved region [SCR]), and a single gene in the complementary-sense known as βC1. The product of the βC1 is a pathogenicity (symptom) determinant, a suppressor of gene silencing (a host defense mechanism triggered by double stranded RNA) and may possibly involved in virus movement [10], [11], [12]. Frequently associated with begomovirus-betasatellite are a second class of small (∼1380 nt) ssDNA molecules known as alphasatellite (previously known as DNA 1; [6]). These are best described as satellite-like, since they encode a rolling-circle replication initiator protein (Rep) and consequently are capable of autonomous replication in plant cells. However, in common with betasatellites, they require the helper begomovirus for movement in and between plants. The precise function of alphasatellites remains unclear, although recent evidence suggest that the Rep they encode is a suppressor of RNA silencing; thus possibly involved in overcoming host defenses [13].

The begomovirus-betasatellite complexes that cause CLCuD in cotton in Asia and Africa are distinct. The complex in Pakistan and India during the 1990s was shown to consists of multiple begomovirus species (often occurring as multiple infectionsmore than one virus per plant) supporting a disease specific betasatellite (Cotton leaf curl Multan betasatellite [CLCuMB]) as well as an alphasatellite [3], [14], [15], [16]. The resistance breaking strain of CLCuD has been shown to consist of a single begomovirus species (a recombinant species derived from two species that were involved in the earlier epidemic) supporting a recombinant CLCuMB but apparently lacking an alphasatellite [17], [18]. The disease in Africa is believed to have been caused by a single begomovirus (*Cotton leaf curl Gezira virus* [CLCuGV]) and a disease specific betasatellite (Cotton leaf curl Gezira betasatellite [CLCuG]); the presence of an alphasatellite has thus far not been investigated [4], [5]. Southern Pakistan (Sindh Province) has in the past not been severely affected by CLCuD. However, in 2003 extensive losses occurred and the disease was shown to be associated with a new recombinant species of begomovirus associated with CLCuMB and various alphasatellites [19]. During a routine survey of infected cotton fields in Sindh Province during 2005 we identified the African CLCuD-associated begomovirus, CLCuGV, in symptomatic cotton plants. The significance of this finding to cotton production in Asia and globally is discussed.

## Results

### Identification of an African virus associated with CLCuD in cotton from Sindh, Pakistan

**Figure 1:**
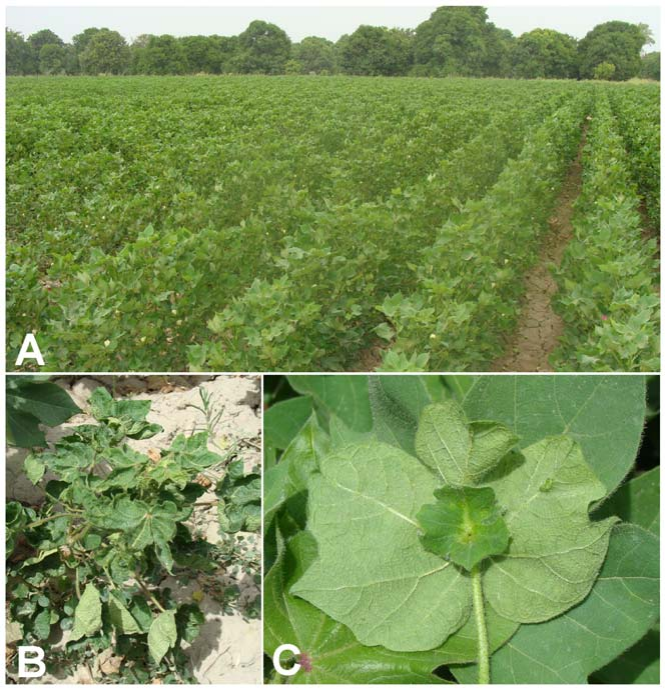
Symptoms of cotton leaf curl disease affected cotton in Sindh, Pakistan. A field of cotton in Sindh showing near complete infection (A). Close up of a severely infected cotton plant showing leaf curling and stunting (B). Underside of a cotton leaf from an infected plant showing vein swelling, enations and leaf-like growths (C).

Leaf samples were collected from 16 cotton plants exhibiting clear CLCuD symptoms in the vicinity of Hala and Tando Adam, Sindh province, in 2005 (Figure 1). Earlier analysis showed all plants to be associated with begomoviruses (results not shown) and the sequencing of one begomovirus isolate showed it to be *Cotton leaf curl Kokhran virus* (CLCuKoV [19]). However, from the remaining samples, PCR amplification using universal primers, designed to amplify all Asian begomoviruses, did not yield a product from five samples. All circular DNA molecules were then amplified from these five samples using rolling circle amplification (RCA) and begomovirus genomes were cloned in a plasmid vector following *Bam*HI restriction of the concatameric RCA product. The complete sequences of five clones (NT1, NT7, NT26, NT28, NT31), one from each plant, were determined and are available in the nucleotide sequence database under the accession numbers FR751142 to FR751146 (Table S1).

The sequences are between 2744 and 2764 nt in length, typical of monopartite begomoviruses. Analysis of the sequences show them to encode two genes in the virion-sense (the coat protein [CP] and V2 genes) and four in the complementary-sense (the replication associated protein [Rep], the C2 gene, the replication enhancer [REn] gene and the C4 gene); the positions and coding capacities of these are indicated in Table S1. Comparisons of the five sequences showed them to have between 98.3–99.8% nucleotide sequence identity, showing them to be isolates of a single begomovirus species (the species demarcation threshold for begomoviruses being 89% nucleotide sequence identity [8]).

An initial comparison to the sequences available in the databases using BLAST (NCBI) showed the clones from Pakistan to have the highest levels of sequence identity to CLCuGV. Subsequent sequence alignment using MegAlign (Lasergene sequence analysis package, DNASTAR) showed them to have between 87 and 98.3% nucleotide sequence identity with CLCuGV sequences available in the databases (37 of which are available at this time). To *Hollyhock leaf crumple virus* (HoLCrV), another *Malvaceae*-infecting begomovirus from Africa, the levels of identity were between 81.9 and 83.4%. To all other begomoviruses in the database the levels of sequence identity were less than 77%. These results indicate that the virus identified in association with CLCuD-affected cotton in Sindh Province is CLCuGV. CLCuGV is a typical African begomovirus, distinct from those that occur on the sub-Continent, and is the first such virus identified in Pakistan.

### CLCuGV identified in Sindh likely originates from North Africa

**Figure 2:**
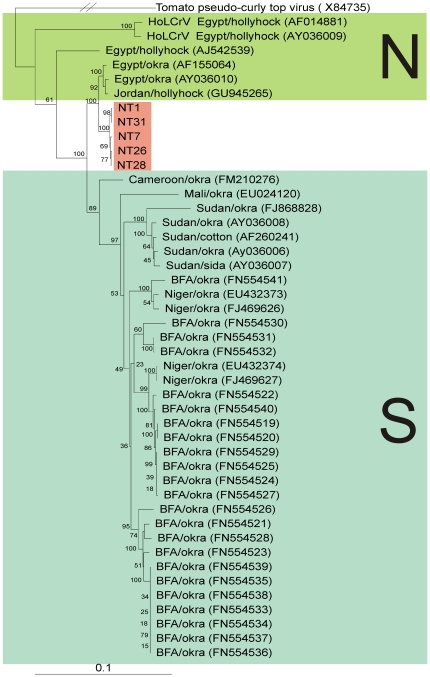
The begomovirus affecting cotton in Sindh likely originates from Egypt. Neigbour-joining phylogenetic dendrogram based upon an alignment of all complete genome nucleotide sequences of *Cotton leaf curl Gezira virus* (CLCuGV) available in the databases and the two available sequences of *Hollyhock leaf crumple virus* (the virus species most closely related to CLCuGV) with the sequences isolated from Pakistan. In each case the database accession number is given. The numbers at nodes represent percentage bootstrap confidence scores (1000 replicates). The alignment was arbitrarily rooted on outgroup, the sequence of *Tomato pseudo curly top virus* (TPCTV), a distantly related geminivirus. The plant species from which viruses were isolated and their geographical origins are indicated; Burkina Faso (BFA). Additionally, the viruses originating from north Africa (N) and from Africa south of the Sahara (S) are indicated.

In an effort to try and identify the possible origins of the CLCuGV identified in southern Pakistan, all available CLCuGV sequences were aligned and used in a phylogenetic analysis (Figure 2). At this time three strains of CLCuGV are recognized, the “Hollyhock”, “Egypt” and “Sudan” strains [8]. The dendrogram shows the CLCuGV sequences originating from Pakistan to segregate with isolates of the “Egypt” strain of CLCuGV and not those of the “Sudan” strain. These three strains show phylogeographic segregation, the “Hollyhock” and “Egypt” strains occurring in North Africa (specifically Egypt and, more recently, Jordan) and the “Sudan” strain occurring in central Africa, south of the Sahara. It is likely the obstacle caused by the Sahara to whitefly migration that has led to divergence of these two strains from their common ancestor. This finding suggests that the CLCuGV identified in Pakistan has its origins in North Africa, which is supported by the finding that CLCuGV originating from Pakistan has the highest levels of sequence identity to isolates of the “Egypt” strain (Table 1). Overall the Pakistani isolates show the highest levels of nucleotide sequence identity (98.1–98.4%) to CLCuGV-Egypt[Egypt:Aswan:Okra] (AF155064), isolated from okra, but lower levels of identity (97.9–98.2%) to an isolate of CLCuGV from hollyhock from Jordan. This possibly pinpoints Egypt as the original source of CLCuGV in Pakistan.

**Table 1:** Percentage nucleotide sequence identities of begomovirus clones isolated from Sindh with *Malvaceae*-infecting viruses from Africa.

Isolate	CLCuGV#			HoLCrV (2)*
	Hollyhock (1)*	Sudan (33)*	Egypt (4)*	
NT1	91.7	87.9–94.2	95.2–98.3	82.182.2
NT7	91.8	87.9–94.2	95.1–98.2	82.182.3
NT26	91.7	88.0–94.2	95.2–98.3	82.082.2
NT28	92.7	87.0–93.3	94.8–98.4	83.283.4
NT31	92.1	87.6–94.1	95.0–98.1	81.982.1

# Three strains of CLCuGV are recognized; the Hollyhock, Sudan and Egypt strains.

*The figures in brackets indicate the numbers of sequences available in the databases for comparison.

### Association of two distinct Asian betasatellites with CLCuGV in Pakistan

In contrast to CLCuMB in Asia, which associates with numerous begomoviruses, CLCuGV in Africa has only ever been identified in association with CLCuGB, the only *Malvaceae*-infecting betasatellite identified on the continent. It is for this reason that efforts were made to identify CLCuGB in the cotton plants in which CLCuGV was found. Despite extensive efforts, that included Southern blotting probing with a CLCuGB probe and RFLP mapping RCA products (results not shown), only two species of betasatellite were identified and both are of Asian origin. The presence of CLCuMB was shown by Southern blotting with a CLCuMB probe and PCR amplification with CLCuMB βC1 gene-specific primers (Figure S1).

The second betasatellite was PCR amplified with betasatellite-specific primers [20], cloned and sequenced. The sequence is 1389 nts in length and available in the sequence databases under accession number FR751147. This sequence is in all respects typical of betasatellites, having a sequence rich in adenine (coordinates 598 to 1100), a SCR (coordinates 1300 to 13) that encompasses a predicted hairpin structure containing, within the loop, the sequence TAATATTAC and a single open reading frame in the complementary-sense conserved between betasatellites (known as the βC1 gene; coordinates 650 to 204) with the capacity to encode a 149 amino acid protein.

**Figure 3:**
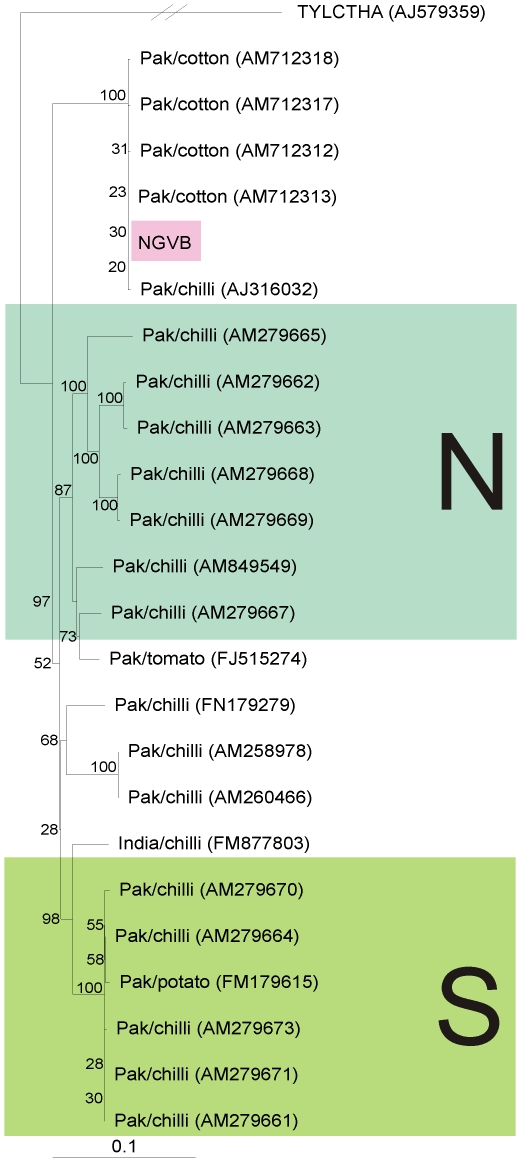
The Chilli leaf curl betasatellite detected in cotton is distinct from the majority of ChLCB detected in other plant species. Neigbour-joining phylogenetic dendrogram based upon an alignment of all full-length nucleotide sequences of ChLCuB available in databases with that isolated from cotton in Sindh (NGVB). In each case the database accession number is given. The numbers at nodes represent percentage bootstrap confidence scores (1000 replicates). The alignment was arbitrarily rooted on outgroup, the sequence of Tomato yellow leaf curl Thailand alphasatellite (TYLCTHA), an unrelated molecule of a similar size. The plant species from which betasatellites were isolated and their geographical origins are indicated. The northern (N) and southern (S) “strains” of ChLCB identified by Hussain et al. [34] are indicated.

Comparison of the sequence of the betasatellite clone (designated NGVB) isolated from cotton with all betasatellites in the databases showed it to have the highest sequence identity (84.9 to 100%) to isolates of Chilli leaf curl betasatellite (ChLCB) but only low levels of identity (<77%) to all other betasatellites (with the highest levels, 76.7%, to an isolate of Tomato leaf curl Bangladesh betasatellite [acc. no. AY438558]) indicating that it is an isolate of ChLCB (the proposed species demarcation threshold for betasatellites being 78% [21]). A phylogenetic analysis of NGVB, based upon an alignment with all ChLCB sequences available in the databases (23 available at this time), is shown in Figure 3. This shows NGVB to segregate with ChLCB isolates recently isolated from cotton in Pakistan [22] and a single ChLCB isolated from chilli pepper (*Capsicum* spp.; AJ316032; [23]). An extensive analysis of the diversity of ChLCB, which showed there to be two geographically distinct variants of this betasatellites, concluded that AJ316032 was unusual. The segregation of AJ316032 with ChLCBs isolated from cotton may indicate that this was a cotton-adapted variant infecting chilli pepper, since the ChLCBs isolate from cotton are distinct from the others isolated from chilli peppers.

These findings thus indicate that CLCuGV in Pakistan is associated with CLCuMB and ChLCB, both having their origins in Pakistan, and that the African betasatellite, CLCuGB, is not present. Additionally, this is the first time CLCuGV has been identified in association with any betasatellite other than CLCuGB. All attempts to identify a possible alphasatellite associated with CLCuGV infections, using alphasatellite-specific primers in PCR and Southern blotting with washing at low stringency, were negative. However, similar analyses of plants not containing CLCuGV showed some, but not all, to habour alphasatellites, including a previously unidentified species [19].

### Infectivity of CLCuGV and associated satellites to *N. benthamiana*

**Figure 4:**
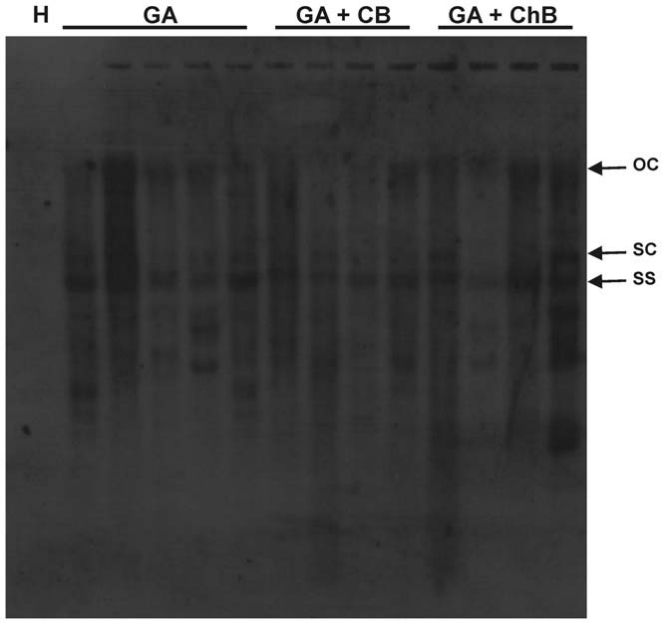
CLCuGV from Sindh replicates in the presence of both CLCuMB and ChLCB in *Nicotiana benthamiana*. Southern blot analysis of DNA extracted from *N. benthamiana* probed for the presence of CLCuGV. Samples were extracted from a healthy plant (H) and plants inoculated with CLCuGV (GA), CLCuGV and CLCuMB (GA+CB) and CLCuGV and ChLCB (GA+ChB). The positions of open circular (oc), supercoiled (sc) and single stranded (ss) viral DNA forms are indicated. Approximately 10 µg of DNA was loaded in each well.

**Figure 5:**
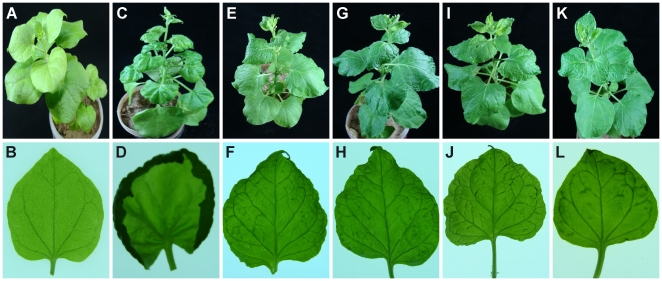
Symptoms induced by virus and betasatellite clones in *Nicotiana benthamiana*. Shown are pictures of plants (upper panels) and close-up views of the undersides of leaves using transmitted light (lower panels). Shown are a healthy plant (panels A and B) and plants inoculated with CLCuMV and CLCuMB (panels C and D), CLCuGV (panels E and F), CLCuGV and ChLCB (panels G and H), CLCuGV and CLCuMB (panels I and J) and CLCuGV, ChLCB and CLCuMB (panels K and L).

To assess the biological competence of the CLCuGV in presence of Asian betasatellite, these were introduced into *N. benthamiana* plants by *Agrobacterium*-mediated inoculation. As a positive control, CLCuMV with CLCuMB were inoculated. This combination was highly infectious and induced typical symptoms of infection, consisting of leaf curling, vein darkening and enations (Figure 1 panels C and D), phenocopying the symptoms of CLCuD in cotton, within 15 days of inoculation (Table 2). Similarly, all inoculations involving CLCuGV were efficient, all plants becoming infected (Table 2), but in contrast to CLCuMV/CLCuMB, symptoms did not appear until 25 days post-inoculation. This may suggest that CLCuGV is poorly adapted to *N. benthamiana*, a suggestion that is supported by the fact that all *N. benthamiana* plants infected with CLCuGV produced copious amounts of defective sub-genomic DNA molecules, in addition to the typical replicative forms of the viral genome (Figure 4). Inoculation of CLCuGV, in the absence of a betasatellite induced relatively mild symptoms consisting of mild stunting, mild leaf curl and vein darkening but no enations (Figure 5, panels E and F). Inoculation of *N. benthamiana* with CLCuGV with ChLCB induced symptoms that did not differ significantly from those induced by CLCuGV alone (Figure 5, panel G and H). In the presence of CLCuMB, the symptoms of CLCuGV infection were very similar to those induce by CLCuGV alone but additionally included enations (Figure 5, panel I and J). However, in contrast to infections of CLCuMB in the presence of CLCuMV, there was only mild leaf curling. In the presence of both betasatellites, the symptoms induced were again similar to those induced by CLCuGV alone, with only mild leaf curling but there was no evidence of enations. For all plants the presence of the inoculated components, both virus and betasatellites, was confirmed by PCR diagnostics (results not shown). Southern blot analysis of infected plants, probed with a CLCuGV probe, showed that the virus DNA levels were not significantly affected by the presence of the betasatellites (Figure 4). These results show that CLCuGV is able to interact with and maintain two distinct betasatellites *in planta.*

**Table 2:** Infectivity of CLCuGV in the presence of betasatellites to *Nicotiana benthamiana*.

Inoculum	Infectivity (no. of plants infected/no. of plants inoculated)		
	Expt. 1	Expt. 2	Expt. 3
CLCuGV	6/6	5/5	10/10
CLCuGV+ChLCB	-	6/6	10/10
CLCuGV+CLCuMB	6/6	5/5	10/10
CLCuGV+ChLCB+CLCuMB	-	6/6	10/10
CLCuMV+CLCuMB	5/5	5/5	5/5

## Discussion

In contrast to all other cotton producing areas of Pakistan, cotton production in Sindh province has only sporadically been affected by CLCuD and even then at only a low incidence. The reasons for this are unclear but a difference in the biotype of the begomovirus vector, *B. tabaci*, has been suggested as a possible factor in this [24]. Nevertheless, in 2003 cotton in Sindh succumbed to infection and has been affected with increasing severity since. Recently we showed that CLCuKoV, one of the earliest begomoviruses shown to be associated with CLCuD in Pakistan [14], [25] and a new recombinant species, *Cotton leaf curl Shahdadpur virus*, together with CLCuMB are associated with the outbreak of CLCuD in Sindh. Here we show that additionally an African virus, CLCuGV, is involved.

**Figure 6:**
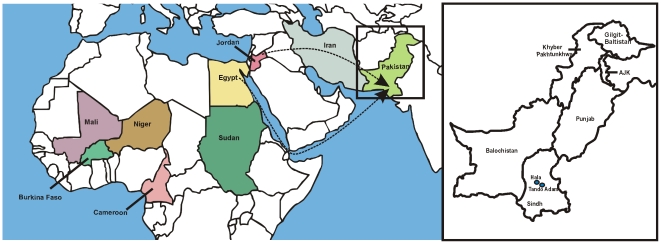
Geographic distribution of CLCuGV and possible routes for spread from Africa to Asia. Countries from which sequences of CLCuGV are available are coloured. Only a small amount of sequence of CLCuGV, forming part of a recombinant *Tomato yellow leaf curl virus* isolate, is available from Iran. The separate map of Pakistan shows Hala and Tando Adam, in Sindh province, from which the CLCuGV clones were obtained. The dashed lines show the possible overland (via the Middle East) and sea routes for the introduction of CLCuGV from North Africa to Pakistan.

The begomoviruses occurring in the Middle East and the Indian sub-Continent are distinct. This is likely due to the geographical hurdle to dispersal posed by the Sulaiman mountain range, which runs down the eastern edge of Balochistan, separating Pakistan from the Iranian plateau. Similarly, the begomoviruses of China and the sub-Continent are distinct, most likely due to the barrier posed by the Himalayan mountain range. This has led to the unusual situation with the monopartite begomovirus *Tomato yellow leaf curl virus* (TYLCV), which has its origins in the Middle East, but has since spread globally, including to the New World, North Africa and much of Asia, but does not occur in either Pakistan or India [26]. Nevertheless, although begomoviruses do not appear to have spread eastwards from Iran into Pakistan, there is evidence for spread westwards into Iran; *Tomato leaf curl Palampur virus* having been reported from Iran, Pakistan and India [27], [28]. Similarly, *Squash leaf curl China virus* has been introduced from China into both Pakistan and India [29]. The question thus arises of how CLCuGV comes to be present in southern Pakistan. Although there are ancient trade routes between North Africa and the sub-Continent, both overland and by sea, the very high levels of sequence similarity suggest that the introduction has occurred quite recently from North Africa, rather than from more southern regions; the virus identified in Pakistan being of the “Egypt” strain rather than the “Sudan” strain. A recent overland introduction is supported by the identification of CLCuGV in Jordan (acc. no. GU945265) and the identification of a recombinant fragment of CLCuGV sequence in a TYLCV isolate from Iran (Figure 6)[26]. Evidence for a more direct introduction of CLCuGV is somewhat more tenuous. We have previously shown that *Bean yellow dwarf virus* (BeYDV), a mastrevirus that was first reported from southern Africa [30], [31], also occurs in Pakistan and likely originates from this region [32]. The introduction of BeYDV to Africa was proposed to have occurred by the migration of peoples from the sub-Continent; many southern and East African countries have sizeable expatriate south Asian communities. It is interesting to note that the “Egypt” strain of CLCuGV has not previously been identified in cotton, instead having only been found in the food crop okra and the ornamental plant hollyhock (*Althea rosea*). As well as indicating that the “Egypt” strain is a threat to cultivated cotton, this provides a possible way for CLCuGV to be transported by peoplein infected ornamental plants.

Only two betasatellites are known to be associated with CLCuD, CLCuMB in Asia and CLCuGB in Africa [3], [4]. The βC1 gene encoded by CLCuMB has been shown to be the major pathogenicity (symptom) determinant of the Asian begomovirus/betasatellite complex, which has the capacity to induce typical disease symptoms in the absence of all other begomovirus-encoded factors when expressed from a *Potato virus X* vector [33]. It is for this reason that cotton plants exhibit essentially the same symptoms irrespective of which begomovirus the plant is infected with; CLCuMB being essential for these viruses to efficiently infect and induce leaf curl symptoms in cotton [3], [14]. Our analyses were unable to identify CLCuGB in plants in which CLCuGV was shown to be present, suggesting either that CLCuGB was not introduced with its cognate begomovirus or that this betasatellite was not maintained; possibly not being able to compete with the native CLCuMB which may be better adapted to local (Asian) cotton varieties. However, since relatively few plants were examined, further efforts will need to be made to confirm the absence of CLCuGB.

Although CLCuGB was not identified, the presence of the Asian CLCuD associated betasatellite CLCuMB was shown. This is the first documented case of CLCuD being caused by interaction between an African virus and an Asian betasatellite. Additionally a non-malvaceous betasatellite, ChLCB, was shown to be present in plants containing CLCuGV and CLCuMB. Recently ChLCB has been encountered increasingly in cotton having been shown to be present in an unusual collection of cotton species in Multan [22]. The significance of this is unclear. ChLCB is a Pakistani betasatellite that appears to have recently (2009) spread into India (acc. no. FM877803) and had, before the study of Azhar et al. [22], only been identified in chilli peppers, potato and tomato. The extensive study of Hussain et al. [34] concluded that there were two “strains” of ChLCB (North and South) in Pakistan. However, one unusual isolate (acc. no. AJ316032) was identified which did not fit the geographical segregation of all other isolates. This ChLCB was found to segregate with the cotton-isolated ChLCB sequences presented by Azhar et al. [22] and segregates with the ChLCB characterized here. This finding may explain the unusual behaviour of AJ316032these may represent cotton-adapted variants of ChLCB. Nevertheless, it is evident that that ChLCB is unable to induce CLCuD without CLCuMB, since in the study of Azhar et al. [22] and here, all plants additionally contained the CLCuD betasatellite CLCuMB. The infectivity studies conducted in *N. benthamiana* suggest that ChLCB may modulate symptoms, since in the presence of both satellites the vein darkening, vein swelling and enation symptoms were more pronounced and occurred more towards the leaf margins than in infections of CLCuGV with either betasatellite alone.

The encroachment of the African CLCuD-associated begomovirus CLCuGV into cultivated cotton in Pakistan is a worrying development. Southern Asia, and particularly Pakistan, already had a large genetic diversity of begomovirues capable of infecting cotton. It is likely this diversity, in both cotton and other plant species, added to the rapidity with which resistance to CLCuD in cotton was broken after it was introduced in the late 1990s. The possible effects of the added genetic diversity of an African virus remain to be determined. However, the evidence of recombination between CLCuGV and TYLCV, a distantly related virus that is not known to infect plants of the family *Malvaceae* (including cotton), in Iran indicates that genetic exchange between even distantly related viruses can occur [26]; a phenomenon that likely occurs in common weed hosts. Possibly the only saving grace is that the African CLCuD-associated betasatellite (CLCuGB) appears not to be present in Pakistan at this time. CLCuMB and CLCuGB are the only betasatellites known to be able to induce CLCuD in cotton at this time. However, more extensive field surveys have been scheduled to assess the validity of this conclusion, which is based upon only limited evidence.

CLCuD is a potential threat to all cotton growing regions of the World since these are all areas where the begomovirus vector *B. tabaci* occurs. In the United States cotton is affected by a bipartite begomovirus, *Cotton leaf crumple virus* (CLCrV; [35]) which is distinct from the Asian and African CLCuD-associated viruses. CLCrV is a minor problem since it usually infects plants late in the growing season and thus does not cause significant losses. Cotton cultivation in China has, until recently, remained free of CLCuD. However, in 2008 CLCuD was reported from Guangxi province and shown to be associated with CLCuMV and CLCuMB, indicating that the Asian begomovirus-betasatellite complex has spread [36]. Cotton cultivation in Australia is free of CLCuD. However, Saeed [37] has shown that a virus native to Australia, and causing problems in tomato crops [38], has the capacity to interact with CLCuMB and transiently infect cotton, inducing CLCuD symptoms. There is thus the possibility that, were CLCuMB introduced into Australia, this could affect cotton production. Taken together with the findings presented here, this indicates that the viruses and satellites that cause CLCuD can spread considerable distances and have the ability to interact with local viruses and possibly affect cotton. Further monitoring is required to ascertain the precise spread of the viruses and to devise effective measures to prevent further spread which may include stricter controls on the movement of agricultural products and particularly the international trade in ornamental plants.

## Materials and Methods

### Plant sample collection and DNA extraction

Leaves of cotton plants showing typical CLCuD symptoms were collected from Hala, Sanghar, Shahdadpur and Tando Adam, in Sindh province Pakistan, in 2005. Total genomic DNA was extracted from symptomatic leaves by using the CTAB method [39] and stored at −20°C.

### Rolling circle amplification

Rolling circle amplification (RCA) using ϕ29 DNA polymerase was used to amplify all circular DNA molecules in DNA samples [40], [41]. For this a reaction mixture of 20 µl, containing 100 to 200 ng of genomic DNA, 1 mM dNTPs, 50 µM random hexamer primers, 2 µl 10X ϕ29 DNA polymerase reaction buffer, was prepared and incubated at 94°C for 3 minutes to denature double stranded DNA. Then the mixture was cooled to room temperature and 5–7 units of Ф29 DNA polymerase (Fermentas, Arlington, Canada) and 0.02 units of pyrophosphatase were added and incubated at 30°C for 18 hours. After that Ф29 DNA polymerase was inactivated at 65°C for 10 minutes.

### Cloning and sequencing

Concatameric RCA product was digested independently with various restriction endonucleases to identify enzymes yielded fragments of begomovirus genome (∼2.8 kb) or half genome size (∼1.4 kb). *Bam*HI yielded approx 2.8 kb fragment and this was ligated into the pTZ57R vector (Fermentas, Arlington, Canada). No restriction enzymes yielding approx 1.4 kb fragments could be identified, so potential betasatellites were PCR-amplified from RCA amplified product with the universal primers Beta01/Beta02 [20]. A PCR product of expected size was ligated into the plasmid vector pTZ57R/T using an InsT/A cloning kit (Fermentas, Arlington, Canada). Similarly, primers DNA101/102 [42] were used in PCR to amplify possible alphasatellites. Selected clones were sequenced commercially (Macrogen, Korea).

### DNA sequence and phylogenetic analysis

The Basic Local Alignment Search Tool (BLAST, NCBI) was used to compare sequences with begomovirus and betasatellite sequences in the database. Sequence alignments of full length clones with the various references sequences were constructed using CLUSTAL X [43]. Phylogenetic trees were generated from aligned sequences using the neighbor-joining method with bootstrapping (1000 replicates). Trees were viewed and manipulated using Treeview [44].

### Production of constructs for *Agrobacterium*-mediated inoculation

Partial direct repeat constructs of virus and satellite clones were produced for *Agrobacterium*-mediated inoculation. An ∼437 bp *Bam*HI-*Pst*I fragment of the begomovirus clone NT1 was ligated in the binary vector pGreen0029 [45] to generate pGNT0.15. Then full-length *Bam*HI insert of NT1 was ligated into *Bam*HI restricted pGNT0.15 to yield a 1.15-mer construct of the viral genome (pGNT1.15). Similarly, to generate a 1.6-mer of the betasatellite clone NGVB, a fragment of ∼859 bp was released by digestion with *Kpn*I and *Eco*RI and cloned into pGreen0029 to yield pGNGB0.6. The full-length betasatellite was released from pTZ57R/T by digestion with *Kpn*I and ligated into *Kpn*I restricted pGNGB0.6 to yield pGNGB1.6. Both constructs were electroporated into *Agrobacterium tumefaciens* strain GV3101 and inoculated to *N. benthamiana* plants as previously described [46]. Plants were maintained in an insect-free glasshouse at 25°C with supplementary lighting to give a 16 hour day length and examined daily for symptoms of virus infection. The presence of betasatellites in plants was detected by PCR with specific primers to amplify the CLCuMB βC1 gene [33] or the ChLCB βC1 gene (CβC1FTATGAATCGATATGCACCACGTATATGAA/CβC1RATACTGTCGACTCACACACACACATTCGTAC).

## Supporting Information

Figure S1 **Detection of Cotton leaf curl Multan betasatellite (CLCuMB) in CLCuD affected cotton plants from Sindh.** Southern blot analysis of nucleic acids extracted from field collected cotton plants probed for the presence of CLCuMB (A). Samples were extracted from a healthy, glasshouse grown, cotton plant and from three of the five cotton plants in which CLCuGV was detected (lanes 2–4). The blot was probed with the βC1 gene of CLCuMB. PCR-mediated detection of CLCuMB using primers specific for the CLCuMB βC1 gene (B). The template DNA included in the PCR reactions were extracted from a healthy, glasshouse grown, cotton plant (lane 1), from an *N. benthamiana* plant experimentally infected with CLCuMV and CLCuMB (lane 2) and from the 5 plants with CLCuD symptoms originating from Sindh in which CLCuGV was detected (Halalanes 3–6 and Tando Adamlane 7). PCR products were run on a 1% agarose gel and stained with ethidium bromide. A size marker was run in lane M.
(TIF)

Table S1 Genes encode by CLCuGV clones isolated from Pakistan. * The Rep gene of NT31 contains a frame shift mutation due to the insertion of an A (with respect to the other isolates) at coordinate 2179. The data in the table is for a reconstructed Rep gene product.
(DOCX)
