# Supplementary figures and images for: The Merging of Two Dynasties—Identification of an African Cotton Leaf Curl Disease-Associated Begomovirus with Cotton in Pakistan
Source: PLoS One. 2011 May 26;6(5):e20366. doi: 10.1371/journal.pone.0020366 (PMC3102712; doi:10.1371/journal.pone.0020366)

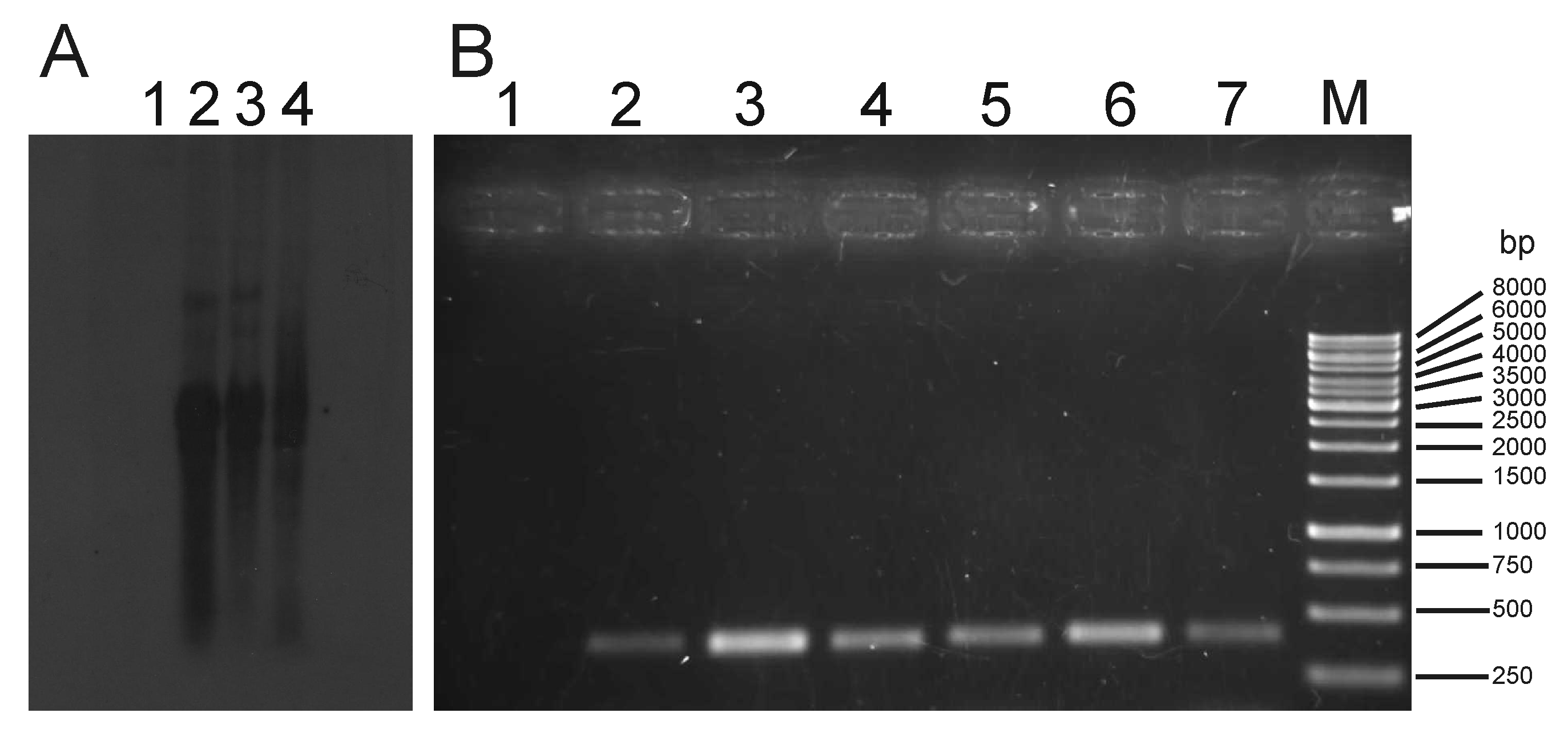

Supplement: Figure S1 — Detection of Cotton leaf curl Multan betasatellite (CLCuMB) in CLCuD affected cotton plants from Sindh. Southern blot analysis of nucleic acids extracted from field collected cotton plants probed for the presence of CLCuMB (A). Samples were extracted from a healthy, glasshouse grown, cotton plant and from three of the five cotton plants in which CLCuGV was detected (lanes 2–4). The blot was probed with the βC1 gene of CLCuMB. PCR-mediated detection of CLCuMB using primers specific for the CLCuMB βC1 gene (B). The template DNA included in the PCR reactions were extracted from a healthy, glasshouse grown, cotton plant (lane 1), from an N. benthamiana plant experimentally infected with CLCuMV and CLCuMB (lane 2) and from the 5 plants with CLCuD symptoms originating from Sindh in which CLCuGV was detected (Halalanes 3–6 and Tando Adamlane 7). PCR products were run on a 1% agarose gel and stained with ethidium bromide. A size marker was run in lane M. (TIF) [file pone.0020366.s001.tif]
