# Supplementary material for: The Merging of Two Dynasties—Identification of an African Cotton Leaf Curl Disease-Associated Begomovirus with Cotton in Pakistan
Source: PLoS One. 2011 May 26;6(5):e20366. doi: 10.1371/journal.pone.0020366 (PMC3102712; doi:10.1371/journal.pone.0020366)
Supplement: Table S1 — Genes encode by CLCuGV clones isolated from Pakistan. * The Rep gene of NT31 contains a frame shift mutation due to the insertion of an A (with respect to the other isolates) at coordinate 2179. The data in the table is for a reconstructed Rep gene product. (DOCX) [file pone.0020366.s002.docx]

**Table S1**. Genes encode by CLCuGV clones isolated from Pakistan.

| Isolate [isolate descriptor] | Accession number | Origin | Size (bp) | Genes (coordinates/coding capacity [number of amino acids]) | | | | | |
| --- | --- | --- | --- | --- | --- | --- | --- | --- | --- |
|  |  |  |  | CP | V2 | Rep | C2 | REn | C4 |
| NT1[Pak:NT1:05] | FR751142 | Hala | 2763 | 322-1098/**258** | 162-530/**122** | 1544-2632/362 | 1240-1644/**134** | 1095-1496/**133** | 2182-2475/**97** |
| NT7[Pak:Hala:05] | FR751143 | Hala | 2763 | 322-1098/**258** | 162-530/**122** | 1544-2632/362 | 1240-1644/**134** | 1095-1496/**133** | 2182-2475/**97** |
| NT26[Pak:NT26:05] | FR751144 | Hala | 2763 | 322-1098/**258** | 162-530/**122** | 1544-2632/362 | 1240-1644/**134** | 1095-1496/**133** | 2182-2475/**97** |
| NT28[Pak:NT28:05] | FR751146 | Tando Adam | 2744 | 303-1079/**258** | 143-511/**122** | 1525-2613/**362** | 1221- 1625/**134** | 1076-1477/**133** | 2163-2456/**97** |
| NT31[Pak:NT31:05] | FR751145 | Hala | 2764 | 322-1098/**258** | 162-530/**122** | 1544-**2633/362*** | 1240-1644/**134** | 1095-1496/**133** | 2183-2476/**97** |

* The Rep gene of NT31 contains a frame shift mutation due to the insertion of an A (with respect to the other isolates) at coordinate 2179. The data in the table is for a reconstructed Rep gene product.
